# Novellierung der Musterweiterbildungsordnung 2026

**DOI:** 10.1007/s00393-026-01868-4

**Published:** 2026-09-25

**Authors:** Alexander Pfeil, Anna Voormann, Martin Aringer, Isabell Haase, Ina Kötter, Gabriela Riemekasten, Ulf Wagner, Torsten Witte, Martin Fleck, Christof Specker

**Affiliations:** 1 Rheumazentrum (nach G-BA-Regeln) sowie Sektion Rheumatologie und Osteologie, Klinik für Innere Medizin III, Universitätsklinikum Jena, Friedrich-Schiller-Universität Jena, Am Klinikum 1, 07747 Jena, Deutschland; 2 Deutsche Gesellschaft für Rheumatologie und Klinische Immunologie e. V., Berlin, Deutschland; 3 Bereich Rheumatologie, Medizinische Klinik und Poliklinik III, Universitätsklinikum „Carl Gustav Carus“ der Technischen Universität Dresden, Dresden, Deutschland; 4 Sektion für Rheumatologie und Entzündliche Systemerkrankungen, Universitätsklinikum Hamburg Eppendorf (UKE), Hamburg, Deutschland; 5 Klinik für Rheumatologie und klinische Immunologie, Universitätsklinikum Schleswig-Holstein, Campus Lübeck, Lübeck, Deutschland; 6 Bereich Rheumatologie, Klinik und Poliklinik für Endokrinologie, Nephrologie, Rheumatologie, Universitätsklinikum Leipzig, Leipzig, Deutschland; 7 Klinik für Rheumatologie und Immunologie, Medizinische Hochschule Hannover, Hannover, Deutschland; 8 Klinik und Poliklinik für Innere Medizin I, Universitätsklinikum Regensburg, Regenburg, Deutschland; 9 Klinik und Poliklinik für Rheumatologie/Klinische Immunologie, Asklepios Klinikum Bad Abbach, Bad Abbach, Deutschland; 10 Düsseldorf, Deutschland

**Keywords:** Weiterbildung, Musterweiterbildungsordnung, Kompetenzen, Continuing education, Model training regulations, Competences

## Abstract

Die Novellierung der Musterweiterbildungsordnung (MWBO) für die Fachärztin bzw. den Facharzt für Innere Medizin und Rheumatologie erfolgt vor dem Hintergrund tiefgreifender Strukturveränderungen im Deutschen Gesundheitssystem. Die Versorgung wird sich grundlegend ändern; stationär durch das Krankenhausversorgungsverbesserungsgesetz (KHVVG) und das Krankenhausreformanpassungsgesetz (KHAG), ambulant durch das vorgesehene Primärarztsystem – und alle „Anbieter“ von Gesundheitsleitungen sind mit dem GKV-Beitragssatzstabilisierungsgesetz (BStabG) konfrontiert. Im Rahmen des 130. Deutschen Ärztetags wurde eine Novellierung der MWBO hinsichtlich der Weiterbildungszeiten vorgenommen. Die neue MWBO hält an der Gesamtweiterbildungszeit von mindestens 72 Monaten für Innere Medizin mit Schwerpunkt fest, reduziert jedoch die verpflichtende stationäre Weiterbildungszeit im Schwerpunkt von 24 auf 18 Monate. In diesem Kontext analysiert der Beitrag, wie die Novellierung der MWBO eine Facharztweiterbildung in der Rheumatologie gewährleisten kann, die qualitativ hochwertig und zukunftsfähig ist.

## Abstract (en)

The revision of the model special training regulations (*Musterweiterbildungsordnung*, MWBO), the federal template for training of specialists in internal medicine and rheumatology, occurs in the context of significant structural changes in the German healthcare system. Medical care structures will change, for inpatient care based on the Hospital Care Improvement Act (KHVVG) and the Hospital Reform Adjustment Act (KHAG), for outpatient care based on changes towards the primary care providers and all caregivers will have to adapt to the Health Insurance Premium Stabilization Act (BStabG). At the 130th German Federal Medical Assembly, an amendment was adopted for the MWBO with respect to training periods. The new MWBO maintains the total training period of 72 months for all internal medicine subspecialties but reduces the mandatory inpatient training period in the specialty from 24 to 18 months. In this context, the present article analyzes how the amended MWBO can ensure specialist training in rheumatology that is of high quality and fit for the future.

Die Novellierung der Musterweiterbildungsordnung (MWBO) 2026 für den Facharzt bzw. die Fachärztin für Innere Medizin und Rheumatologie markiert einen Einschnitt von fachlicher sowie gesundheitspolitischer Relevanz. Die vorliegende Arbeit beleuchtet die Entwicklung der neuen MWBO für die Innere Medizin und Rheumatologie.

## Bisherige Gliederung der Weiterbildung

Gemäß der bisherigen MWBO 2018 basierte die Facharztausbildung für Innere Medizin auf drei Säulen: Für den Facharzt für Innere Medizin ohne Schwerpunktbezeichnung war bisher eine Weiterbildungszeit von 60 Monaten vorgesehen. Sollte zusätzlich zu dieser Facharztweiterbildung eine Schwerpunktbezeichnung erworben werden, waren zusätzlich 36 Monate Weiterbildungszeit im Schwerpunkt abzuleisten. Dagegen war für die Facharztweiterbildung Innere Medizin und Schwerpunkt eine Weiterbildungszeit von insgesamt 72 Monaten vorgesehen. Für diese Facharztbezeichnung waren gemäß der geltenden Bestimmungen jeweils mindestens 36 Monate für die internistische Basisweiterbildung und für das Schwerpunktfach, z. B. „Rheumatologie“, vorgesehen. Innerhalb des Schwerpunktfachs waren bislang mindestens 24 Monate in der stationären Patientenversorgung verpflichtend zu absolvieren, während zwölf Monate Weiterbildungszeit im Schwerpunkt auch in der ambulanten Patientenversorgung erfolgen können [1].

Dieses Modell hatte den Vorteil, dass zentrale klinische Kompetenzen im Umgang mit akuten Schüben bei entzündlich-rheumatischen Erkrankungen (ERE), Organmanifestationen, schweren Infektionen unter Immunsuppression und komplexen immunmodulatorischen Therapien regelhaft im stationären klinischen Alltag erworben werden konnten [4].

## Novellierung der Musterweiterbildungsordnung 2026

Vor dem Hintergrund des Krankenhausversorgungsverbesserungsgesetzes (KHVVG) und des Krankenhausreformanpassungsgesetzes (KHAG) wurde im Rahmen des 130. Deutschen Ärztetags (DÄT) in Hannover vom 12. bis 15. Mai 2026 ein Konsensvorschlag zur Novellierung der MWBO für die Fachärztin/den Facharzt für Innere Medizin mit und ohne Schwerpunkt verabschiedet. Dabei wird die Weiterbildungszeit für die Internistin/den Internisten ohne Schwerpunktbezeichnung unverändert bei 60 Monaten belassen, und die Weiterbildungszeit für den zusätzlichen Erwerb eines Schwerpunktes für internistische Fachärztinnen und Fachärzte von 36 Monate auf 24 Monate verkürzt. Nach intensiver Diskussion beschloss der DÄT eine unveränderte Weiterbildungszeit für den Facharzt „Innere Medizin und Schwerpunkt“ von 72 Monaten. Dabei entfallen weiterhin 36 Monate auf eine „Basisweiterbildung“ in allgemeiner Innerer Medizin. Darin sind verpflichtend sechs Monate internistische Intensivmedizin, sechs Monate Weiterbildung in der internistischen Notaufnahme sowie 24 Monate in mindestens zwei internistischen Fachgebieten vorgesehen, die außerhalb des angestrebten Schwerpunktbereichs liegen.

**Abb. 1:**
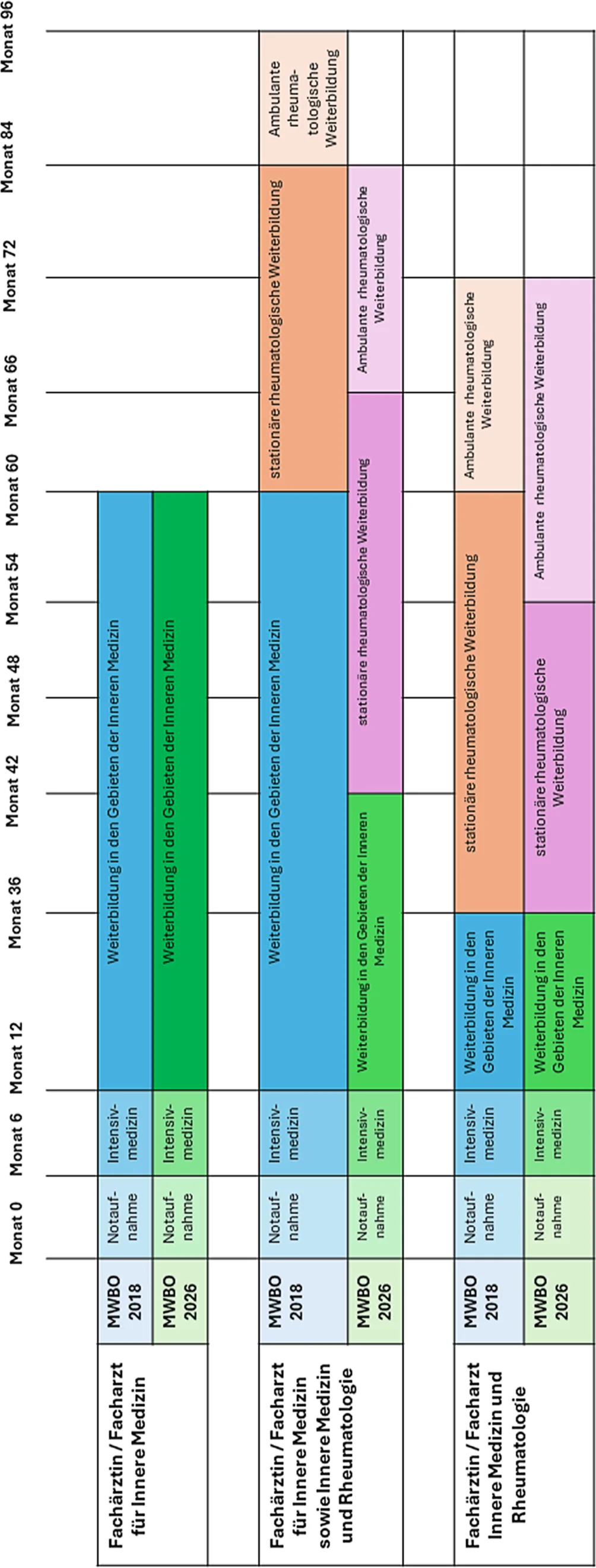
Übersicht zu den Mindestweiterbildungszeiten entsprechend der Novellierung der Musterweiterbildungsordnung (MWBO) 2026 im Vergleich zur Musterweiterbildungsordnung 2018 für die Fachärztin/Facharzt für Innere Medizin, Doppelfachärztin/Facharzt für Innere Medizin sowie Innere Medizin und Rheumatologie als auch Fachärztin/Facharzt für Innere Medizin und Rheumatologie

Darüber hinaus sind 36 Monate Weiterbildung im Schwerpunkt vorgesehen. Hier wird aber die bisherige obligatorische stationäre Weiterbildungszeit im Schwerpunkt von 24 auf 18 Monate reduziert. Dieser Vorschlag wurde von der Deutschen Gesellschaft für Innere Medizin, den internistischen Schwerpunktgesellschaften und dem Berufsverband Deutscher Internistinnen und Internisten erarbeitet und nach intensiven Diskussionen und Abstimmungen konsentiert. Der Zeitraum von 18 Monaten entsprach dabei dem Mehrheitsvotum der Schwerpunktgesellschaften, welche sich für eine stationäre Mindestweiterbildungszeit im Schwerpunkt zwischen 12 und 24 Monaten ausgesprochen hatten (Abb. 1).

## Mögliche Konsequenzen für die Rheumatologie aus der neuen Musterweiterbildungsordnung 2026

Gemäß der Novellierung der MWBO 2026 ermöglicht es die Weiterbildung zum Facharzt für Innere Medizin und Rheumatologie nun, einen längeren Weiterbildungsabschnitt (18 Monate) im ambulanten Sektor zu absolvieren. In diesem Bereich erfolgt ein signifikanter Anteil der Langzeitbetreuung entzündlich-rheumatischer Erkrankungen. Diese Änderung entspricht dem gesundheitspolitischen Ziel einer sektorenübergreifenden, kompetenzorientierten Weiterbildung. Darüber hinaus eröffnet es die Möglichkeit, den Erwartungen vieler Weiterzubildender gerecht zu werden, die ausdrücklich an ambulanten Ausbildungsabschnitten und an kombinierten klinisch-ambulanten Berufsbildern interessiert sind [7]. Eine durch erhöhte Flexibilität gekennzeichnete Weiterbildungsstruktur kann zudem die Vereinbarkeit von Beruf und Familie sowie moderne Teilzeit- und Kooperationsmodelle verbessern [7]. Dies gewinnt vor dem Hintergrund des demografischen Wandels, auch in der Ärzteschaft, zusätzlich an Bedeutung [2].

So überzeugend die stärkere Einbindung ambulanter Strukturen ist, so klar müssen zugleich die Limitationen benannt werden. Nicht alle Weiterbildungsinhalte lassen sich ambulant in der erforderlichen Tiefe erwerben. Dies betrifft insbesondere akute und potenziell lebensbedrohliche Verläufe entzündlich-rheumatischer Systemerkrankungen, die eine 24/7-Aufnahmebereitschaft, engmaschige Überwachung, invasive Diagnostik und komplexe Therapieentscheidungen erfordern, aber oft auch ein eng abgestimmtes und situativ angepasstes interdisziplinäres Vorgehen.

Die überwiegende Mehrzahl der Facharztweiterbildungen (Weiterbildungsstellen [gesamt] *n* = 478,4; Krankenhausbereich 81,8 % versus Niederlassung 18,2 %) in der Rheumatologie findet im stationären Bereich statt [5]. Dabei bildet die akutstationäre Rheumatologie eine essenzielle Säule der Weiterbildung. Entsprechend der MWBO 2018 § 2a Absatz 4 wird die stationäre Akutversorgung wie folgt definiert: „Stationäre Akutversorgung zeichnet sich aus durch eine stationäre Behandlung von Patienten aus einem unausgelesenen Patientenkollektiv in Bezug auf Diagnosen und Altersstruktur, die wegen einer akuten, unvorhergesehenen Erkrankung in einem Krankenhaus mit einer Aufnahmebereitschaft von 24 h an sieben Tagen in der Woche aufgenommen werden“ [1].

In diesem Umfeld erfolgt der Erwerb jener Kompetenzen, die für die Versorgung schwerer Kollagenosen und Vaskulitiden, autoinflammatorischer Syndrome, primärer und sekundärer Immundefekte sowie schwerer Infektionen unter Immunmodulation – bis hin zu lebensbedrohlichen Fällen – von grundlegender Bedeutung sind [4].

Darüber hinaus existieren Patientenkollektive, deren Altersstruktur, Multimorbidität, funktionelle Einschränkung oder psychosoziale Belastung eine stationäre Versorgung zwingend erforderlich machen und die im ambulanten Setting nur unzureichend abgebildet werden können [4].

## Einordnung der Novellierung der Musterweiterbildungsordnung 2026 in den gesundheitspolitischen Kontext

Im niedergelassenen Bereich sind 43,1 % Weiterbildungsstellen nicht besetzt. Daher bietet die Ausweitung ambulanter Weiterbildung eine gute Möglichkeit [5], die Zahl von Fachärztinnen und Fachärzten auf Dauer zu erhöhen. Um beim Ausschöpfen dieses Potenzials die Qualität der Weiterbildung auf hohem Niveau zu halten, muss definiert werden, welche fachlichen Kompetenzen in der ambulanten Regelversorgung vermittelt werden können und welche allein im stationären Umfeld erlernbar sind. Dabei ist zu betonen, dass zu den ERE weit mehr Krankheitsbilder zählen als die Erkrankungen des Bewegungsapparates. Sie umfassen eine Vielfalt von komplexen immunologischen Systemerkrankungen mit potenzieller Multiorganbeteiligung. Für die Vermittlung der notwendigen Kompetenzen und die Nutzung ambulanter Weiterbildungsangebote bieten sich somit aufeinander abgestimmte, kombinierte und sich ergänzende Weiterbildungsmodelle in ambulanten und stationären Versorgungseinrichtungen an.

## Weiterentwicklung des Mustercurriculums und des Positionspapiers zur Weiterbildungsbefugnis

Unabhängig davon, welche Kompetenzen in der ambulanten und welche in der stationären Patientenversorgung in welchen Mindestweiterbildungszeiten vermittelt werden können, soll sich die MWBO auf die inhaltliche Ausgestaltung fokussieren. Dies impliziert eine zunehmende Verantwortung der Fachgesellschaften, die fachspezifischen Teile der MWBO und des fachlich empfohlenen Weiterbildungsplans (FEWP) konkret zu definieren. Auch setzt dies eine Anpassung der Inhalte an die Weiterbildungsbefugnis der Weiterbildungseinrichtung voraus, die zudem überprüfbar sein sollen. Nur so lässt sich bei größerer Flexibilität eine qualitativ hochwertige strukturierte Weiterbildung gewährleisten. Für das Fach Rheumatologie liegen mit dem Mustercurriculum der Deutschen Gesellschaft für Rheumatologie und Klinische Immunologie (DGRh) und dem Positionspapier zur Weiterbildungsbefugnis bereits zwei Instrumente vor [3,6 ], die diese Aufgabe inhaltlich und strukturell abbilden.

Das Mustercurriculum ist darauf ausgerichtet, den Erwerb von Kompetenzen über einen Zeitraum von drei Ausbildungsjahren zu strukturieren und eine Adaption an unterschiedliche Weiterbildungsorte zu ermöglichen [6]. Das Positionspapier zur Weiterbildungsbefugnis fungiert als Ergänzung zu diesem Konzept, insofern es Mindestanforderungen an Weiterbildungsstätten definiert sowie eine gestufte Vergabe der Weiterbildungsbefugnis über 12, 24 oder 36 Monate festlegt [3]. Im Rahmen der MWBO-Novellierung erlangt dieser Kriterienkatalog hohe Relevanz, da er eine entscheidende Verbindung zwischen Flexibilisierung und Qualitätssicherung herstellt.

## Aufgaben der Landesärztekammern

Auf die Landesärztekammern kommt nun zunächst die Ratifizierung der neuen MWBO für den jeweiligen Kammerbereich zu. Des Weiteren ist die Rolle der Landesärztekammern bei der Erteilung der Weiterbildungsbefugnisse von besonderer Bedeutung. Im Zuge einer zukünftigen Entwicklung, die eine verstärkte Implementierung der Weiterbildung in ambulanten, stationären bzw. sektorübergreifenden Strukturen mit sich bringt, wird eine kompetenzbasierte Evaluation erforderlich, um zu ermitteln, welche Inhalte eine Weiterbildungsstätte tatsächlich vermitteln kann. Die neue Logik der Weiterbildung verlangt demnach eine enge Kopplung von Befugnisumfang, vorhandenen Kompetenzen, Kooperationen und realen Leistungszahlen.

Die von der DGRh vorgeschlagenen Kriterien zur Erteilung der Weiterbildungsbefugnis bieten hierfür ein praktikables Raster [3]. Sie ermöglichen Transparenz bei der Vergabe der Weiterbildungsbefugnis, fördern Verbundweiterbildungen über stationäre und ambulante Sektoren hinweg und machen fehlende Kompetenzbereiche sichtbar, die durch Kooperationen ausgeglichen werden können [3]. Auch in Hinblick auf die Krankenhausreform könnte dies ein wichtiger Baustein sein, um einen Rückgang stationärer Weiterbildungskapazitäten zu vermeiden.

## Resümee

Die Novellierung der MWBO 2026 stellt die Rheumatologie vor eine gestalterische Gesamtaufgabe. Diese Entwicklung ist auf die Verlagerung von Versorgungsleistungen in den ambulanten Bereich sowie eine zukünftig sektorenübergreifende Weiterbildung im Rahmen der Ambulanten Spezialfachärztlichen Versorgung (ASV) und der Rheumazentren (Regeln des Gemeinsamen Bundesausschusses [G-BA]) zurückzuführen. Gleichzeitig wird betont, dass stationäre Lernorte auch perspektivisch eine entscheidende Grundlage für eine hochwertige rheumatologische Facharztweiterbildung bilden. Die Zukunft liegt nicht in einem Gegensatz von ambulant und stationär, sondern in einer intelligenten Verzahnung beider Sektoren. Eine zeitgemäße rheumatologische Weiterbildung muss ambulante Langzeitversorgung, ASV und Zentrenstrukturen systematisch integrieren und gleichzeitig die stationäre Akutversorgung als integralen Bestandteil bewahren. Die Novellierung der MWBO kann nur unter dieser Voraussetzung den Anspruch erfüllen, bei der Reaktion auf die veränderte Versorgungslandschaft die Qualität der Weiterbildung und die Kompetenz des Faches zu erhalten.

## Fazit für die Praxis

- Die Novellierung der MWBO ist auch eine Reaktion auf gesundheitspolitische Entwicklungen, wie die Krankenhausreform, die Ambulantisierung und die sektorenübergreifende Versorgung. Sie eröffnet neue Spielräume für ambulante Weiterbildungsanteile.
- Für das Fach Rheumatologie bleibt die stationäre Weiterbildung auch in Zukunft von entscheidender Bedeutung, weil wesentliche Kompetenzen in den Bereichen schwerer Systemerkrankungen, Organmanifestationen, Infektionen und komplexer Immuntherapien nicht in vollem Umfang und Tiefe in der ambulanten Versorgung vermittelt werden können.
- Das Mustercurriculum sowie die Kriterien zur Weiterbildungsbefugnis stellen bereits heute geeignete Instrumente dar, um die neue Weiterbildungsstruktur kompetenzbasiert und qualitätsgesichert zu gestalten.

## Data Availability

Alle dieser Arbeit zugrunde liegenden Daten sind in diesem Artikel enthalten.

## Abkürzungen

ASV: Ambulant-spezialfachärztliche Versorgung
DÄT: Deutscher Ärztetag
DGRh: Deutsche Gesellschaft für Rheumatologie und klinische Immunologie
FEWP: Fachlich empfohlener Weiterbildungsplan
G‑BA: Gemeinsamer Bundesausschuss
MWBO: Musterweiterbildungsordnung
